# Parent experiences of diabetes care questionnaire (PEQ-DC): reliability and validity following a national survey in Norway

**DOI:** 10.1186/s12913-018-3591-y

**Published:** 2018-10-12

**Authors:** Hilde Hestad Iversen, Ylva Helland, Oyvind Bjertnaes, Torild Skrivarhaug

**Affiliations:** 1 Norwegian Institute of Public Health, PO Box 4404, Nydalen, N-0403 Oslo, Norway; 2 Norwegian Directorate of Health, PO Box 7000, N-0130 Oslo, Norway; 3 Division of Paediatric and Adolescent Medicine, The Norwegian Childhood Diabetes Registry, Oslo University Hospital, PO Box 4956, Nydalen, N-0424 Oslo, Norway; 4 Institute of Clinical Medicine, University of Oslo, N-0318 Oslo, Norway

**Keywords:** Questionnaire, Diabetes, Parent satisfaction, Parent experience, Reliability, Validity

## Abstract

**Background:**

Patient experiences are acknowledged as an important aspect of health care quality but no validated instruments have been identified for the measurement of either parent or patient experiences with outpatient paediatric diabetes care. The aim of the current study was to assess the psychometric properties of a new instrument developed to measure parent experiences of paediatric diabetes care at hospital outpatient departments in Norway.

**Methods:**

The development of the questionnaire was based on a literature review of existing questionnaires, qualitative interviews with both parents and children/adolescents, expert-group consultations, pretesting of the questionnaire and a pilot study. The national pilot study included parents of 2606 children/adolescents aged 0–17 years with Type 1 Diabetes registered in The Norwegian Childhood Diabetes Registry, a nationwide, population-based registry. Levels of missing data, ceiling effects, factor structure, internal consistency, item discriminant validity and construct validity were assessed.

**Results:**

A total of 2606 patients were included in the survey, but 80 were excluded due to incorrect addresses. 1399 (55%) parents responded to the questionnaire. Low levels of missing or “not applicable” responses were found for 31 of the 35 items (< 10%), and 27 of 35 items were below the ceiling-effect criterion. Psychometric testing and theoretical considerations identified six scales: Consultation (six items), organisation (five items), equipment (three items), nurse contact (four items), doctor contact (four items) and outcome (five items). All six scales met the 0.7 criterion for Cronbach’s alpha (range: 0.71–0.90). As expected, each item had a higher correlation with its hypothesised scale than with any of the other five scales. The construct validity of the Parent Experiences of Diabetes Care Questionnaire (PEQ-DC) was supported by 17 out of 18 associations with variables expected to be related to parent experiences.

**Conclusion:**

The psychometric testing of the PEQ-DC showed good evidence for data quality, internal consistency and construct validity. The instrument includes important aspects of diabetes care at paediatric outpatient departments from the perspective of the parent. The content validity of the PEQ-DC was secured by a rigorous development process, and the instrument was tested following a national survey in Norway, securing generalisability across Norway.

## Background

In the last few decades, patient satisfaction and patient experiences have drawn increasing interest and have been acknowledged as a critical component of quality assessment. Consistent associations have been found between patient experiences and clinical effectiveness and patient safety [1]. The importance of incorporating patients’ needs and perspectives into care delivery has been highlighted, but when it comes to children under a certain age, parents often act as a proxy response. The information provided by patients or parents or caregivers can be used to inform quality improvement initiatives and improve services [2, 3].

The prevalence of Type 1 Diabetes (T1D) is increasing worldwide, but the prevalence varies between countries. Norway has one of the highest incidences of childhood onset T1D in the world [4, 5], but recent data show that the rise in incidence may have levelled off [5]. According to calculations from the Norwegian Prescription Database, around 28,000 people (0.6% of the population) have T1D [6].

High quality of care is essential to avoid acute and long-term complications [7]. In Norway, children have regular follow-up appointments at the local outpatient diabetes clinics every third months. If the treatment goals are not met, or there are other concerns, the consultation frequency is increased. Before designing the content and structure of the questionnaire we asked the different paediatric departments for information on how often the consultations only include a nurse or only a doctor, how often they include both, and how often patients have consultations with other patients (group consultations). 20 of 27 departments responded, and the feedback showed that the organisation of the consultations differed. Some departments always provide consultations with both a paediatrician and a diabetes nurse participating. At other departments the patient always sees the paediatrician alone, but are most of the times met by the diabetes nurse. Other departments have arranged for the patient to see the paediatrician and the diabetes nurse at every second appointment. In one department, due to staffing problems, the patients only meet the paediatrician once a year. Some of the clinics also provides age matched group consultation for patients in addition to individual consultations. Dieticians and psychologists can be consulted if requested. The degree of parental involvement is essential to disease management [8].

T1D is a demanding chronic disease and caring for a child with T1D has been described as an overwhelming experience, requiring constant vigilance [9, 10]. Type 1 diabetes usually develops in childhood or adolescence. Accordingly, parents play an important role in the day-to-day disease management of both pre-adolescent children and adolescents, and this responsibility places considerable demands on parents [11, 12]. The diabetes management can only be effective if the caregiver or family and the patient are able to implement it. Family involvement is a crucial component of optimal management throughout childhood and adolescence [13].

Parents of children with T1D prefer health care tailored to the individual needs of parents and child, for instance, to the developmental level of the child and to their history regarding the disease [12]. The need for active emotional and practical support from professionals is the greatest in the period immediately after diagnosis [11]. After this the needs are more related to support in aberrant situations and upon request [12]. Parents want to be guided through the enormous amount of information available on the Internet, information about ongoing trials and study results, but also guidance on how this information can be applied to everyday diabetes management. The accessibility of health care professionals is also emphasised [11, 12], as well as the benefits of continuity of care [14]. Parents of older children want opportunities to speak to health professionals alone because young children could be distracting and/or the parents would prefer not to raise distressing issues in front of their child [14].

To secure the quality and usefulness of studies of patient or parent experiences, valid and reliable measures are crucial. Instruments have been developed to characterise the transition experiences of emerging adults with T1D [15]. However, a review of the literature identified no available psychometrically sound instruments that addressed parent satisfaction with paediatric diabetes care at outpatient hospital departments. Consequently, a new measure was developed.

The aim of the present study was to assess the data quality, internal consistency reliability and validity of the Parent Experiences of Diabetes Care Questionnaire (PEQ-DC). The PEQ-DC was developed and evaluated in accordance with the standard methodology of the national patient experience survey programme in Norway [16–29], also supported by criteria that have been defined as important in the measurement of patient satisfaction [30]. The questionnaire was designed for application in a national survey of parents or primary caregivers with children/adolescents aged 0–17 years with T1D included in The Norwegian Childhood Diabetes Registry (NCDR) attending paediatric outpatient departments.

## Methods

### Questionnaire development and content

The development of the PEQ-DC was based on a process including several standardised steps carried out to ensure sound psychometric properties. A literature review was conducted to identify relevant questionnaires, but also to explore methodological issues relevant when addressing health care for children and adolescents with diabetes. An expert group was established comprising health personnel, researchers and representatives from patient organisations. We decided against the use of different versions for various age groups to ensure the questionnaire could be used for parents of children and adolescents with T1D of all ages. Semi-structured interviews were undertaken with 14 parents of children/adolescents aged 5 to 17 years who had received care from outpatient clinics. The interviews were undertaken by two senior researchers independently. The content of the first draft of the questionnaire was informed by the review of the literature, the qualitative interviews and consultations and meetings with the expert group. The first draft was pretested by cognitive interviews with 15 parents of children aged 4 to 17 years, and minor changes were made to the questionnaire before the national pilot survey.

The instrument tested in the pilot survey comprised 40 questions. We asked the parents about their experiences with the paediatric outpatient clinic where their child has diabetes controls. The questionnaire was divided into sections addressing questions about reception and waiting, organisation, nursing services, doctor services, the consultation, parent issues, information and training, access to care and usefulness. The questionnaire also included five items concerning sociodemographic characteristics. Five-point scales with response options that ranged from “not at all” (1) to a “very large extent” (5) were used for most items relating to the experience of care. Two items had a 5-point response format ranging from 1 “very dissatisfied” to 5 “very satisfied”. One item had a 3-point response scale ranging from 1 to 3; 1 = “too few”, 2 = “appropriate”, 3 = too many. Most items included a “not applicable” option. An open-ended question on the last page probed further comments on experiences with the outpatient clinic or feedback on the questionnaire. An English version of the questionnaire is available in a recent publication from this study [31].

### Data collection

The national survey was conducted by the Norwegian Institute of Public Health (NIPH), commissioned by the NCDR, a nationwide, population-based quality registry.

The questionnaire was tested in a national pilot survey including 2606 parents of children and adolescents aged 0–17 years with T1D registered in the NCDR and with at least one outpatient consultation the previous year. NCDR transferred data about the sample to the NIPH, including contact information as a basis for conducting the survey. Data from 27 paediatric outpatient departments in Norway was collected.

All patients registered in the NCDR were included, and their parents were contacted by mail and sent an invitation letter in November 2016. Non-respondents were sent up to two reminders. The sample was randomised into three groups to compare different data collection methods: Group A was posted questionnaires with only a pen-and-paper response option; group B were posted questionnaires with both an electronic and a pen-and-paper response option and, finally, group C were posted questionnaires with only an electronic response option. The three groups were compared on response rate, background variables about respondents, main study results and survey costs. Results from the randomised comparison are reported in another publication [31].

### Statistical analysis

All 34 questionnaire items that addressed experiences of care and had 5-point response formats were considered relevant to include in initial factor analyses. Items were assessed for levels of missing data, ceiling effect and underlying structure of the items. Items with missing data or responses in the “not applicable” option > 10% were omitted from factor analysis to avoid an extensive loss of responses. Using this criteria for inclusion, 30 out of 34 items were included. A ceiling effect is when marks are clustered in a few boxes at one extreme, in patient experience surveys, often near the very top of the scale, making it almost impossible to detect any improvement or to distinguish between various grades of excellence [32]. The criteria relating to ceiling effects vary between studies. In this study we set the cut off to 50%: If the item should be considered acceptable, fewer than 50% could tick the most favourable response option [27, 28, 33].

Exploratory factor analysis (EFAs) with principal axis factoring was conducted to assess the underlying factor structure of the items. Correlations among the factors were expected, and oblique rotation (promax) was chosen for interpretability of results. The criterion for the number of factors to be rotated was eigenvalues greater than 1. Items with factor loadings lower than 0.4 were excluded, and no cross-loadings over 0.30 were retained. However, the analysis and the subsequent scales were not only data-driven, but also founded on theoretical considerations underpinning the analyses.

The internal consistency reliability of the scales was assessed using item-total correlation and Cronbach’s alpha. Item-total correlation measures the strength of association between the individual item and the remainder of its scale, and a correlation of 0.40 was considered acceptable [34]. An alpha value between 0.70 and 0.90 was considered satisfactory [34–37].

Item discriminant validity was tested by correlating all single items with the scales. Each item was expected to have a significantly higher correlation with its hypothesised scale than with the other scales [38]. The associations between the items and the scales were assessed with Spearman’s rho.

The literature review did not identify any obvious variables for inclusion in the construct validity testing, neither did a literature review on patient satisfaction measurement in general [39], or results from previous parent experience studies in the national survey programme in Norway [19, 22, 40]. We did not have access to patient background data when the current study was carried out. Thus, we had no background information on the child. In the absence of gold standard measures, the construct validity testing adopted an exploratory approach, and background variables assumed to relate to the scales were tested. It was hypothesised that scale scores were associated with parent participation, education and considerations regarding the number of consultations at the paediatric department. Age, gender and whether the respondent was living with the child’s other parent were also explored. Correlations between background questions and the scales were assessed with t-tests and one-way ANOVA for categorical questions and by Spearman’s rank correlations (*r*) for continuous variables.

All statistical analyses were conducted using SPSS version 23.0.

## Results

A total of 2606 patients were included in the survey, but 80 were excluded because of incorrect addresses. 1399 (55%) parents or guardians responded to the questionnaire. Table 1 lists the characteristics of the respondents. The mean age was 43.4 years and 1090 (78.5%), were female. As shown in Table 1, 66.8% had a university or college education and 78.7% were living with the child’s other parent. Of the 1399 parents that responded, 1150 (82.8%) had participated in the consultation three or more times in the last year.

**Table 1:** Sample characteristics (*n* = 1399)

	Number	Percent
Gender		
Male	299	21.5
Female	1090	78.5
Age (years)	1359	43.4 (mean)
Education		
Primary school	43	3.1
Secondary school	418	30.1
University or college (0–4 years)	461	33.2
University or college (4 ≥ years)	466	33.6
Living with the child’s other parent		
Yes	1084	78.7
No	294	21.3
Number of times participated in the consultation during the last year		
None	16	1.2
1	61	4.4
2	162	11.7
3	398	28.7
4 or more	752	54.1

Levels of missing data, responses in the “not applicable” option, mean and ceiling effect for the items are shown in Table 2. Levels of missing data ranged from 0.1 to 4.0%. Responses in the “not applicable” category ranged from 0.4 to 38.3%. Rates of missing and “not applicable” < 10% were considered acceptable, and 31 of the 35 items were retained for further analyses. The lowest mean score on the scale of 1–5, where 5 is the best possible experience, was for the item relating to the child’s access to a psychologist (2.14). The highest score of 4.57 was for the item addressing the doctor’s knowledge of diabetes and diabetes care. The majority of items had a score in the range of 3–4 on the 1–5 scale. Furthermore, 27 out of 35 items were below the ceiling-effect criterion of 50%.

**Table 2:** Item descriptives

		n	Missing (%)	Not applicable (%)	Mean_a_	Ceiling (%)
1	Are you welcomed in a satisfactorily manner when you arrive at the paediatric department?	1392	0.5	–	4.40	48.1
2	Do you think you spend a lot of time waiting at the paediatric department?	1393	0.4	–	3.91	19.5
3	Do you find the waiting room satisfactory?	1389	0.7	–	3.67	13.5
4	Do you think the paediatric department is well organised?	1318	0.5	5.3	4.00	22.4
5	Do you think the doctors and nurses cooperate well with each other?	1360	0.1	2.7	4.25	39.9
6	Do you think the person providing the consultation is well prepared?	1391	0.1	0.5	4.18	38.5
7	Do you and your child meet the same nurses every time you visit the paediatric department?	1392	0.1	0.4	4.38	53.5
8	Do you and your child have sufficient time for contact with the nurses?	1379	0.5	0.9	4.28	43.0
9	Do you think the nurses are knowledgeable about diabetes and diabetes care?	1340	3.7	0.5	4.52	59.6
10	Do you think the nurses are concerned about and care for your child?	1339	3.8	0.5	4.50	58.5
11	Do you and your child meet the same doctor every time you visit the paediatric department?	1324	3.6	1.7	4.36	57.5
12	Do you and your child have sufficient time for contact with the doctor?	1329	3.9	1.1	4.22	43.0
13	Do you think the doctor is knowledgeable about diabetes and diabetes care?	1322	4.0	1.5	4.57	65.3
14	Do you think the doctor is concerned about and cares for your child?	1326	3.8	1.4	4.38	52.9
15	In your opinion, do the issues addressed in the consultation meet your child’s needs?	1338	3.6	0.8	4.22	40.7
16	Is it clear to you and your child what should be followed up before the next consultation?	1328	3.9	1.1	4.07	37.2
17	Are you and your child involved in deciding what to follow up before the next consultation?	1298	4.0	3.2	3.89	30.4
18	Are your views as a parent taken seriously?	1339	3.8	0.5	4.26	43.3
19	Do you have enough time for conversations without your child being present?	1048	3.8	21.3	2.74	12.0
20	Are you given satisfactory information and guidance on how to follow up on your child’s diabetes treatment?	1335	3.7	0.9	4.05	34.1
21	Do you receive the support you need to transfer more of the responsibility for diabetes treatment to your child?	1191	3.8	11.1	3.85	29.4
22	Do you receive satisfactory information on the results of tests and examinations?	1349	3.6	–	3.91	27.8
23	Do you receive satisfactory information on the development in your child’s health and the risk of complications?	1320	3.8	1.9	3.66	23.0
24	Do you receive satisfactory information from the paediatric department on what equipment is available?	1338	3.7	0.6	3.31	13.1
25	Do you and your child receive good training in managing the equipment?	1319	3.4	2.3	4.00	31.3
26	In your opinion, does your child have satisfactory access to the best possible equipment?	1294	3.5	4.0	3.53	21.0
27	Does your child have satisfactory access to a dietitian?	1095	3.5	18.2	2.81	10.3
28	Does your child have satisfactory access to a psychologist?	812	3.6	38.3	2.14	6.4
29	Is it easy to make contact with the paediatric department outside of appointments?	1271	0.9	8.3	3.67	24.2
30	What do you think about the number of consultations at the paediatric department? ^b^	1372	0.9	1.1	1.90	
31	Do you think that your child benefits from visiting the paediatric department?	1381	0.3	1.0	3.92	31.9
32	Do you, as a parent, benefit from visiting the paediatric department?	1383	0.6	0.6	4.12	39.5
33	Does the follow up at the paediatric department make you and your child more capable of having a good life with diabetes?	1383	0.5	0.6	4.06	35.9
34	All in all, how dissatisfied or satisfied are you with the way in which the paediatric department has followed up your child and the diabetes treatment? ^c^	1391	0.6	–	4.35	53.5
35	All in all, how dissatisfied or satisfied are you with the way in which the paediatric department has attended to you as a guardian? ^c^	1391	0.6	–	4.39	54.3

^a^All items were scored on a 5-point response scale ranging from 1 (“not at all”) to 5 (“to a very large degree”)

^b^Item with 3-point response scale ranging from 1 to 3 (1 = “too few”, 2 = “appropriate”, 3 = “too many”)

^c^Items with 5-point response scale ranging from 1 (“very dissatisfied”) to 5 (“very satisfied”)

All 25 items relating to structure and process were included in the first factor analysis. However, after comprehensive testing, we decided to analyse the items addressing nurse and doctor contact separately to render results more useful for the purposes of local improvement. The organisation of outpatient clinics in Norway differs and integration of the nurse and doctor contact items could confuse the interpretability of the results; this assumption was supported by the empirical testing. Factor analysis including the remaining 17 items on structure and process resulted in three factors. Three items (5, 19 and 22) were excluded from the factor analysis, one by one, due to cross loadings > 0.30 and also low factor loadings on own factor (< 0.40). The results suggested three factors with an eigenvalue of > 1 accounting for 64.3% of the total variation: i) consultation ii) organisation and iii) equipment (Table 3). The second factor analysis included the four items addressing nurse contact and produced one factor with an eigenvalue of > 1 explaining 55.7% of the variation. The third factor analysis included the four items concerning doctor contact and produced one factor that explained 62.2% of the variation. Finally, the fourth factor analysis included the five outcome items (31–35) and produced one factor accounting for 69.5% of the variation.

**Table 3:** Factor loadings and reliability statistics

		Factor loadings	Corrected item-total correlation	Cronbach’s alpha
Consultation				0.90
16	Is it clear to you and your child what should be followed up before the next consultation?	0.91	0.78	
17	Are you and your child involved in deciding what to follow up before the next consultation?	0.90	0.72	
15	In your opinion, do the issues addressed in the consultation meet your child’s needs?	0.85	0.76	
18	Are your views as a parent taken seriously?	0.80	0.75	
20	Are you given satisfactory information and guidance on how to follow up on your child’s diabetes treatment?	0.61	0.73	
6	Do you think the person providing the consultation is well prepared?	0.52	0.68	
Organisation				0.71
2	Do you think you spend a lot of time waiting at the paediatric department?	0.64	0.40	
3	Do you find the waiting room satisfactory?	0.61	0.42	
4	Do you think the paediatric department is well organised?	0.59	0.57	
1	Are you welcomed in a satisfactory manner when you arrive at the paediatric department?	0.57	0.55	
29	Is it easy to make contact with the paediatric department outside of appointments?	0.42	0.47	
	Equipment			0.79
24	Do you receive satisfactory information from the paediatric department on what equipment is available?	0.86	0.71	
26	In your opinion, does your child have satisfactory access to the best possible equipment?	0.81	0.60	
25	Do you and your child receive good training in managing the equipment?	0.55	0.62	
Nurse contact^a^				0.73
10	Do you think the nurses are concerned about and care for your child?	0.78	0.59	
9	Do you think the nurses are knowledgeable about diabetes and diabetes care?	0.69	0.53	
8	Do you and your child have sufficient time for contact with the nurses?	0.60	0.53	
7	Do you and your child meet the same nurses every time you visit the paediatric department?	0.49	0.44	
Doctor contact^a^				0.79
14	Do you think the doctor is concerned about and cares for your child?	0.79	0.65	
13	Do you think the doctor is knowledgeable about diabetes and diabetes care?	0.73	0.62	
12	Do you and your child have sufficient time for contact with the doctor?	0.69	0.61	
11	Do you and your child meet the same doctor every time you visit the paediatric department?	0.61	0.54	
Outcome^a^				0.89
33	Does the follow up at the paediatric department make you and your child more capable of having a good life with diabetes?	0.84	0.78	
32	Do you, as a parent, benefit from visiting the paediatric department?	0.80	0.75	
34	All in all, how dissatisfied or satisfied are you with the way in which the paediatric department has followed up your child and the diabetes treatment?	0.80	0.74	
35	All in all, how dissatisfied or satisfied are you with the way in which the paediatric department has attended to you as a guardian?	0.74	0.69	
31	Do you think that your child benefits from visiting the paediatric department?	0.74	0.69	

^a^Separate factor analysis for “Nurse contact”, “Doctor contact” and “Outcome”, respectively

Table 3 shows that the item-total correlations for the final six scales are acceptable, ranging from 0.40 to 0.78. All scales met the criterion of 0.70–0.90 for Cronbach’s alpha, ranging from 0.71 to 0.90.

The results of item discriminant validity testing are shown in Table 4. As expected, all items had a substantially higher correlation with its hypothesised scale than with any of the other scales in the questionnaire. All correlations were statistically significant (*p* < 0.001).

**Table 4:** Correlations between items and scales

		Consultation	Organisation	Equipment	Nurse contact	Doctor contact	Outcome
16	Is it clear to you and your child what should be followed up before the next consultation?	**0.86**	0.45	0.51	0.53	0.60	0.58
17	Are you and your child involved in deciding what to follow up before the next consultation?	**0.83**	0.43	0.45	0.48	0.52	0.52
15	In your opinion, do the issues addressed in the consultation meet your child’s needs?	**0.81**	0.47	0.47	0.54	0.62	0.63
18	Are your views as a parent taken seriously?	**0.81**	0.47	0.48	0.56	0.58	0.62
20	Are you given satisfactory information and guidance on how to follow up on your child’s diabetes treatment?	**0.81**	0.50	0.59	0.53	0.56	0.65
6	Do you think the person providing the consultation is well prepared?	**0.75**	0.54	0.51	0.56	0.56	0.61
2	Do you think you spend a lot of time waiting at the paediatric department?	0.27	**0.61**	0.21	0.29	0.20	0.26
3	Do you find the waiting room satisfactory?	0.30	**0.64**	0.27	0.26	0.26	0.31
4	Do you think the paediatric department is well organised?	0.52	**0.71**	0.44	0.43	0.44	0.51
1	Are you welcomed in a satisfactorily manner when you arrive at the paediatric department?	0.52	**0.71**	0.44	0.47	0.42	0.51
29	Is it easy to make contact with the paediatric department outside of appointments?	0.46	**0.73**	0.46	0.43	0.42	0.47
24	Do you receive satisfactory information from the paediatric department on what equipment is available?	0.58	0.45	**0.89**	0.44	0.44	0.53
26	In your opinion, does your child have satisfactory access to the best possible equipment?	0.41	0.37	**0.83**	0.32	0.34	0.43
25	Do you and your child receive good training in managing the equipment?	0.62	0.52	**0.79**	0.51	0.47	0.57
10	Do you think the nurses are concerned about and care for your child?	0.58	0.46	0.43	**0.75**	0.47	0.54
9	Do you think the nurses are knowledgeable about diabetes and diabetes care?	0.49	0.37	0.39	**0.72**	0.41	0.47
8	Do you and your child have sufficient time for contact with the nurses?	0.53	0.45	0.36	**0.78**	0.48	0.44
7	Do you and your child meet the same nurses every time you visit the paediatric department?	0.32	0.31	0.26	**0.71**	0.30	0.27
14	Do you think the doctor is concerned about and cares for your child?	0.65	0.45	0.45	0.49	**0.79**	0.55
13	Do you think the doctor is knowledgeable about diabetes and diabetes care?	0.54	0.38	0.38	0.41	**0.73**	0.47
12	Do you and your child have sufficient time for contact with the doctor?	0.60	0.42	0.41	0.49	**0.83**	0.47
11	Do you and your child meet the same doctor every time you visit the paediatric department?	0.38	0.29	0.27	0.36	**0.74**	0.31
33	Does the follow up at the paediatric department make you and your child more capable of having a good life with diabetes?	0.61	0.47	0.50	0.48	0.46	**0.84**
32	Do you, as a parent, benefit from visiting the paediatric department?	0.58	0.47	0.44	0.47	0.46	**0.84**
34	All in all, how dissatisfied or satisfied are you with the way in which the paediatric department has followed up your child and the diabetes treatment?	0.65	0.53	0.57	0.53	0.56	**0.79**
35	All in all, how dissatisfied or satisfied are you with the way in which the paediatric department has attended to you as a guardian?	0.63	0.52	0.50	0.50	0.53	**0.76**
31	Do you think that your child benefits from visiting the paediatric department?	0.55	0.42	0.44	0.42	0.41	**0.82**

Correlations in bold show item to own scale correlations

Table 5 shows that the associations between the scale scores and the tested variables were statistically significant in 17 out of 18 tests. Associations with age, gender and if the respondent were living with the child’s other parent were all insignificant or very low and are not reported. Participation in the consultation during the last year was significant for all scales (range 0.15–0.19): The higher the number of times parents had participated in the consultation, the higher the scores were on all six scales on a scale of 0 to 100 where 100 is the best possible experience of care. Associations between education and the six scales were significant for all except one (doctor contact). Higher education was associated with lower scores on all five scales. Significant differences (all at the < 0.001 level) were also found between the scores of parents who considered the number of consultations as too few or too many and those who considered the number of consultations as appropriate (range 9–14 points), with the latter group reporting better experiences.

**Table 5:** Construct validity testing: associations between scales, background variables and responses to individual questions

	Consultation	Organisation	Equipment	Nurse contact	Doctor contact	Outcome
Number of times participated in whole or parts of the consultation during the last year	0.16***	0.13***	0.16***	0.15***	0.14***	0.19***
Education	**	*	***	*	Ns	**
Primary school	85.03	78.31	77.92	88.91	88.28	86.10
Secondary school	79.51	73.96	66.52	86.45	84.82	81.10
University or college (0–4 years)	76.31	72.24	62.94	84.07	83.82	78.23
University or college (4 ≥ years)	77.16	73.35	64.85	85.01	83.81	77.88
What do you think about the number of consultations at the paediatric department?	***	***	***	***	***	***
Too few or too many	66.47	64.45	52.44	78.50	74.91	67.45
Appropriate	79.73	74.84	67.28	86.49	85.88	81.29

***p < 0.001; **p < 0.01; *p < 0.05; ns: not significant. Spearman’s rank correlations (*r)* for continuous variables. One-way ANOVA for “Education” and Independent-Samples T-Test for “What do you think about the number of consultations at the paediatric department?”

## Discussion

This study was the first national survey undertaken to assess parent experiences of paediatric diabetes care at outpatient departments in Norway. The psychometric testing of the PEQ-DC showed good evidence for data quality, internal consistency and construct validity. The content validity of the instrument was secured by a rigorous development process, and the instrument was tested following a national survey, securing generalisability across Norway.

The PEQ-DC is multidimensional and comprised six scales based on both empirical and theoretical assumptions. The results can be used to monitor performance and enable hospitals to identify areas where the parent-perceived quality should be improved. The scales have excellent psychometric properties and are minimally burdensome for patients to complete. The PEQ-DC is also relevant to use as a basis for identifying quality indicators.

The low percentage of omitted answers suggests good acceptability and few responses in the “not applicable” option indicate that the questions are relevant to a majority of the parents. Four items had high levels of “not applicable” responses. The first related to having enough time for conversations without the child present (19), the second to necessary support to transfer more of the responsibility for diabetes treatment to the child (21). The third addresses access to a dietitian, the fourth access to a psychologist. The first and second are probably most relevant for parents with children above a certain age and missing responses to these questions most likely relate to parents of younger children. The probability of parents having such an experience presumably increases with their child’s age. When children grow older and gain more independence and autonomy, they prefer to take more responsibility for their own diabetes management [41]. The third and fourth questions are not important or relevant for all respondents. Those who did not provide a valid answer to these items probably did not have such experiences, and were consequently not able to answer.

We had limited evidence of which variables to include in construct validity testing. Hypotheses based on previous research findings and theory were scarce and the literature review underpinning this work did not identify any relevant questionnaires. A systematic review found a consistent positive relationship between age and satisfaction, but evidence on other variables including gender and education was equivocal [39]. However, the current study does not include the patient, but the parents or caregivers, and the age range is limited (SD = 6.4). The results showed significant associations with the scale scores and participation in the consultation during the last year, education and considerations regarding the number of consultations at the paediatric department, but not with age, gender and whether the respondent was living with the child’s other parent. The variables included self-reported data from the questionnaire, and further tests should also include administrative and clinical variables from the outpatient clinics. However, results from a study in a paediatric cohort of patients with T1D showed that patient and parent satisfaction were not related to other clinical outcome measures [42]. Multiple assessments and analyses are needed to assess whether a construct appears to be valid [32].

The respondents in the current study included a high proportion of women (78.5%). Studies focus less on fathers’ experiences and their role and the influence of their management of T1D than the mothers’ [43, 44]. Research shows that mothers experience more responsibility for diabetes tasks than fathers [10, 44, 45]. Mothers also experience greater perceived burden of medical treatment tasks [46]. Thus, mothers may be more involved in coordinating care and may also have more contact with the paediatric outpatient clinic.

The test-retest reliability was not assessed in this study, but results from previous patient experience surveys in the national programme have found acceptable levels of test-retest reliability for other questionnaires [16–29].

This study addressed the considerations of the parents, not those of the patient. Studies show that while parents’ and their adolescents’ reality perceptions might correlate well, this is not always the case for their views on the degree of importance of different aspects of paediatric diabetes care quality [47], or views and priorities about health and illness [48]. However, parent involvement is necessary until their children are able to manage their diabetes independently. Thus, parents form an integral part of the treatment, and their experiences should be included when assessing the quality of care at the outpatient clinic. Consequently, it is important to understand how parents perceive their child’s health care. Understanding the challenges that parent’s face can inform quality improvement initiatives in outpatient clinics. Both the parents’ and the child’s experiences should be included when assessing the quality of care from the user’s perspective. Paediatricians communicate with both parents and patients and this may require varying levels of information and communication. Health care providers must be capable of evaluating the educational, behavioural, emotional, and psychosocial factors that impact implementation of a treatment plan and work with both the individual and the family to overcome barriers or redefine goals [13].

Methods to enhance response rates should be considered. However, previous studies among parents in the national patient experience survey programme have produced lower or similar response rates of 46% and 54% [19, 22].

### Strengths and limitations

A strength of this study is the large national sample including all paediatric diabetes outpatient clinics in Norway. Also, the study was performed by a third party (the NCDR and the NIPH) not involved in providing health care to the patients. The domains and items were derived from a standardised, comprehensive process. The variables tested in this study included self-reported data from the questionnaire. Further analyses should include administrative and clinical variables from the outpatient clinics.

No background information is available on non-responders. This is a potential bias and future research should undertake more detailed comparisons of respondents and non-respondents to assess the extent of bias.

## Conclusions

The PEQ-DC comprises six scales with excellent internal consistency reliability and validity. The instrument is recommended for future applications designed to access parent experiences of outpatient paediatric clinics in Norway. Further tests of construct validity including clinical data are recommended, to also evaluate the instrument’s usefulness as a basis for external quality indicators.

## Abbreviations

EFA: Exploratory factor analysis
NCDR: The Norwegian Childhood Diabetes Registry
NIPH: The Norwegian Institute of Public Health
PEQ-DC: Parent Experiences of Diabetes Care Questionnaire
T1D: Type 1 Diabetes
